# Successful surgical resection of infective endocarditis involving the left ventricular outflow tract and sigmoid septum

**DOI:** 10.1186/s44215-025-00238-x

**Published:** 2025-12-26

**Authors:** Tomonori Sano, Kazuma Handa, Masaru Ishida, Toshinari Onishi, Keiij Iwata

**Affiliations:** 1 Department of Cardiovascular Surgery, Sakai City Medical Center, Sakai City, Osaka, Japan; 2 Department of Cardiovascular Surgery, The University of Osaka Graduate School of Medicine, Yamada-Oka 2-2, Suita City, Osaka, Japan; 3 Department of Cardiology, Sakai City Medical Center, Sakai City, Osaka, Japan

**Keywords:** Infective endocarditis, Left ventricular outflow tract, Sigmoid septum, Vegetation, Embolic stroke

## Abstract

**Background:**

Vegetation in the left ventricular outflow tract (LVOT) due to infective endocarditis (IE) is extremely rare, and no previous reports have described vegetation on the sigmoid septum. We report a rare case of IE with vegetation attached to the sigmoid septum in the LVOT that was successfully managed by surgical resection.

**Case presentation:**

An 80-year-old woman was admitted with an acute cerebral infarction and fever. Transesophageal echocardiography revealed highly mobile vegetation attached to the sigmoid septum in the LVOT, and blood cultures were positive for Streptococcus agalactiae. Due to the high risk of further embolization, surgical resection was performed using a transaortic approach. The vegetation was easily removed under direct visualization, and the histopathological findings were consistent with IE. The patient’s postoperative course was uneventful, and antibiotic therapy was continued.

**Conclusions:**

Vegetations in the LVOT carry a significant risk of embolic events and potential LVOT obstruction. A transaortic approach allows for safe and effective removal under good visualization. Early surgical intervention should be considered in similar cases.

## Background

Vegetation in infective endocarditis (IE) typically develops on cardiac valves or pacemaker leads, whereas vegetation at other sites is exceedingly rare, accounting for approximately 3% of cases [1]. In particular, involvement of the left ventricular outflow tract (LVOT) is uncommon. Here, we present a rare case of LVOT-IE associated with the sigmoid septum that was successfully managed with surgical resection.

## Case presentation

**Fig. 1:**
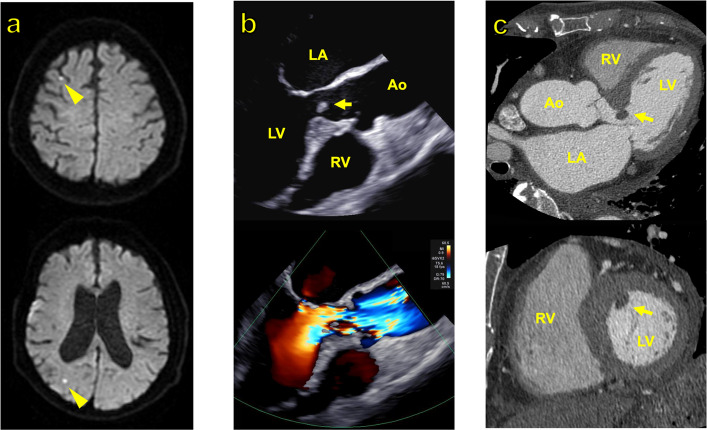
Preoperative imaging findings. **A** Brain MRI at presentation revealed multiple embolic lesions in both the subcortical and cortical areas of the cerebral hemispheres (arrowheads). **B** Preoperative transesophageal echocardiography (long-axis view at 135°) demonstrated a protrusion of the basal interventricular septum into the left ventricular cavity (sigmoid septum), with a mobile mass attached to it. Color Doppler imaging revealed a mosaic turbulent flow pattern in the LVOT, suggestive of systolic flow acceleration. **C** Preoperative contrast-enhanced cardiac CT (long-axis and short-axis views) of the left ventricle clearly visualized the sigmoid septum and the attached mass (arrow)**.** Abbreviations: MRI, magnetic resonance imaging; LVOT, left ventricular outflow tract; CT, computed tomography; LA, left atrium; LV, left ventricle; RV, right ventricle; Ao, aorta

An 80-year-old woman presented with impaired consciousness. Her medical history included paroxysmal atrial fibrillation, cardiogenic cerebral embolism, epilepsy, hypertension, and type 2 diabetes mellitus. Magnetic resonance imaging of the brain revealed multiple acute cerebral infarctions in the cortical and subcortical regions of both cerebral hemispheres (Fig. 1A). The patient’s level of consciousness improved during the clinical course, and no neurological deficits were observed. However, she exhibited fever and elevated inflammatory markers (white blood cell count: 12,650/μL; C-reactive protein: 6.73 mg/dL) and was admitted for further evaluation. Twelve-lead electrocardiography revealed a sinus rhythm at 75 bpm. Plain chest and abdominal computed tomography (CT) revealed no significant findings. Blood cultures were positive for Streptococcus agalactiae. Transthoracic echocardiography showed that the left ventricular end-diastolic/end-systolic diameter was 37/24 mm and the ejection fraction was 67%, with no significant valvular disease detected. However, transesophageal echocardiography revealed a narrow LVOT with systolic flow acceleration due to a sigmoid septum and a 6 × 5 mm mobile vegetation attached to the septum (Fig. 1B). Cardiac CT confirmed the presence of a sigmoid septum and attached vegetation (Fig. 1C), leading to a diagnosis of IE. Antibiotic therapy was initiated with gentamicin (120 mg every 24 h) and ampicillin (2 g every 4 h). Blood culture results were negative after 48 h. However, considering the presence of multiple cerebral infarctions and the highly mobile nature of the LVOT vegetation, the risk of further embolization was deemed high, and surgical intervention was planned.

**Fig. 2:**
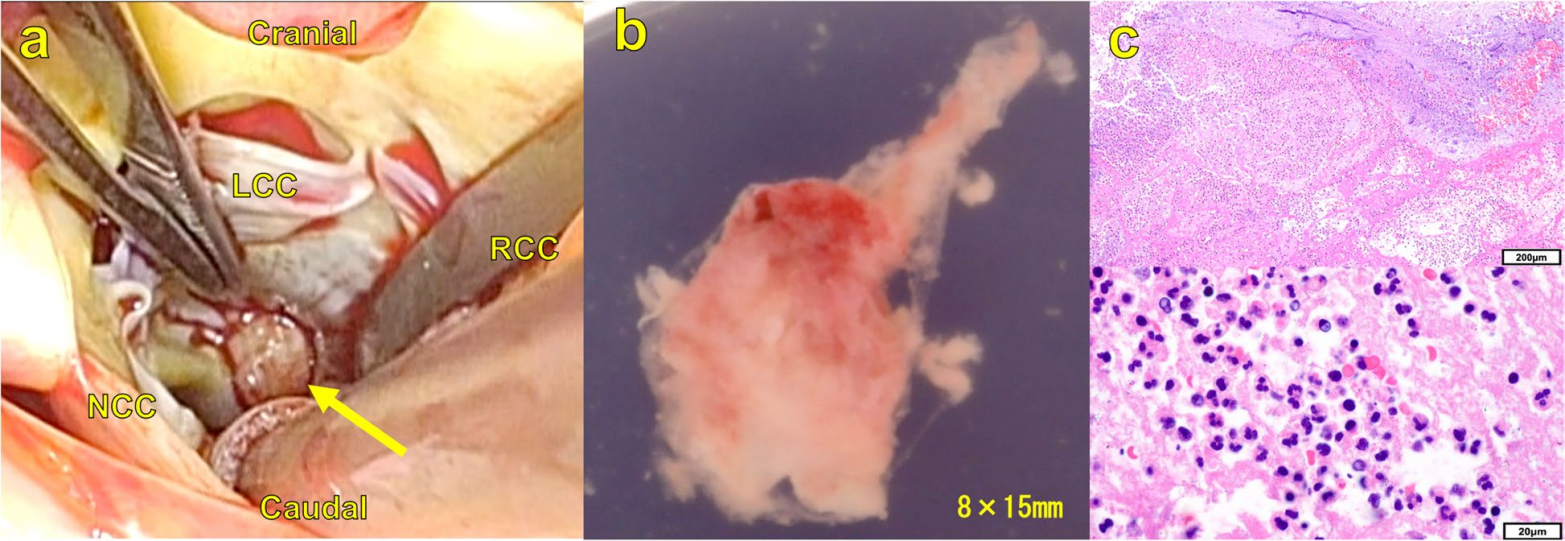
Intraoperative findings and histopathological analysis of the vegetation. **A** Intraoperative transaortic view revealed a tricuspid aortic valve with no abnormalities. A vegetation was identified attached to the LVOT approximately 1 cm below the right coronary cusp (arrow). **B** Excised vegetation specimen. **C** Hematoxylin and eosin staining at low (× 10) and high (× 100) magnification showed inflammatory exudate containing numerous neutrophils. Abbreviations: NCC, noncoronary aortic valve cusp; LCC, left coronary aortic valve cusp; RCC, right coronary aortic valve cusp; LVOT, left ventricular outflow tract

Cardiopulmonary bypass was established via aortobicaval cannulation following median sternotomy. After aortotomy, the transaortic view provided clear visualization of the left ventricular chamber, confirming that the tricuspid aortic valve showed no signs of infection. Inspection of the LVOT revealed vegetation attached approximately 1 cm below the right coronary cusp (Fig. 2A). The vegetation was easily excised (Fig. 2B) and macroscopic endocardial denudation was observed at the attachment site on the sigmoid septum. Partial resection of the sigmoid septum was also performed. Histopathological examination revealed an inflammatory exudate rich in neutrophils but no identifiable bacteria, findings consistent with bacterial vegetation considering the prior initiation of antibiotic therapy (Fig. 2C).

The postoperative course was uneventful. Transthoracic echocardiography confirmed the complete removal of the vegetation and resolution of systolic flow acceleration in the LVOT. The patient received gentamicin for 2 weeks and ampicillin for 4 weeks and was subsequently transferred for further rehabilitation.

## Discussion

The vegetation in IE most commonly involves valvular lesions or pacemaker leads (97%) [1], whereas LVOT involvement is exceedingly rare. To the best of our knowledge, no previous reports have described vegetation formation on the sigmoid septum associated with accelerated flow, as observed herein. In the present case, the vegetation on the sigmoid septum was easily resected using a transaortic approach.

To date, only three case reports of LVOT-IE without underlying structural heart disease, such as valvular disorders, have been published [2–4] (Table 1), and no definitive underlying cause for vegetation formation was identified in any of these cases. Similarly, although our patient lacked valvular disease, the presence of a sigmoid septum may have contributed to vegetation development.

**Table 1:** Comparison of the current case with previous cases of IE with vegetation in the LVOT without coexisting valvular disease

Author	Year	Age	Sex	Complaints	Background	Comorbidity	Site of vegetation	Bacteria	Operation	Embolism	End point
K. Jayaprakash, et al.[2]	2009	33	Male	Fever	-	LVOTO	Septum in LVOT	*Enterococcus faecalis*	-	-	Preoperative death, LVOTO
G. Laguna, et al.[3]	2017	50	Female	Fever, Disturbance of consciousness	CKD on HD	-	Septum in LVOT, PM	MSSA	Resection of the vegetation	Multiple cerebral infarctions	Home, stable condition after 2 months
M. A. Raffali, et al.[4]	2022	50	Female	Fever	-	-	Septum in LVOT	MSSA	Resection of the vegetation	-	Home, stable condition
Current Case	2024	80	Female	Disturbance of consciousness	sigmoid septum	-	Sigmoid septum in LVOT	*Streptococcus agalactiae*	Resection of the vegetation	Multiple cerebral infarctions	Transferred for rehabilitation

*Abbreviations IE* Infective endocarditis, *LVOT* Left ventricular outflow tract, *LVOTO* Left ventricular outflow tract obstruction, *CKD* Chronic kidney disease, *HD* Hemodialysis, *PM* Papillary muscles, *MSSA* Methicillin-susceptible *Staphylococcus aureus*

Key factors in vegetation development include endothelial injury, which leads to thrombus formation that subsequently becomes infected and forms vegetation [5]. Endocardial injury typically results from physical factors such as high-velocity flow or shear stress associated with valvular disease. However, in this case and the three cases summarized in Table 1, the cardiac valves were morphologically and functionally normal, and no physical or hemodynamic factors were identified. Consequently, vegetations were considered to have developed in an atypical location due to mechanisms unrelated to valvular pathology.

In this case, the sigmoid septum likely caused systolic high-velocity flow, which resulted in endocardial injury. During diastole, low-velocity turbulent flow generated by the sigmoid septum may have facilitated bacterial adhesion to the thrombus, resulting in bacterial vegetation. In fact, macroscopic observation during surgery revealed an endocardial defect at the site of vegetation attachment on the sigmoid septum, which was consistent with the above findings.

On the other hand, in the three cases summarized in Table 1, the characteristics of the causative bacteria may have contributed to the formation of vegetation. *Staphylococcus aureus* and *Enterococcus faecalis* were identified as the causative organisms. The former is known for its strong tissue-destructive properties, whereas the latter exhibits high adhesive capacity and biofilm-forming ability [6]. Therefore, even in the absence of overt endocardial injury, these organisms may adhere to minute defects and promote progressive endocardial damage and vegetation formation through their invasive or destructive properties. Additionally, previous reports suggest that hemodynamic instability associated with sepsis may induce secondary endocardial injury and facilitate the development of vegetation [7].

Furthermore, LVOT-IE may be associated with a high embolic potential, although the data are limited owing to its rarity. The reported incidence of embolic complications in IE is 9–17.7% for vegetations on the aortic valve and 17–32.5% for those on the mitral valve [8–10]. In contrast, among the four reported cases of LVOT-IE, including ours, two presented with multiple cerebral infarctions, suggesting a high embolic potential [3]. Furthermore, reports of LVOT-IE causing LVOT obstruction [11] suggest that LVOT-IE may follow a severe clinical course.

Among the four surgically treated LVOT-IE cases, including ours, good visualization and easy vegetation removal were achieved using a transaortic approach. We believe that surgical treatment of LVOT-IE should not be delayed to prevent embolic events and sudden clinical deterioration.

## Conclusion

Herein, we report a rare case of IE with vegetation located in the LVOT, which carries a high risk of embolic events and associated LVOT obstruction. However, they can be safely and effectively resected with good visualization using a transaortic approach. Therefore, early surgical interventions should be considered in similar cases.

## Data Availability

The authors declare that all data in this article are available within the article.

## Abbreviations

CT: Computed tomography
IE: Infective endocarditis
LVOT: Left ventricular outflow tract
